# Time-resolved miRNA-mRNA integrated analysis reveals the miRNA-mRNA networks underlying plasma membrane damage-dependent senescence and DNA damage response-dependent senescence in WI-38 normal human fibroblasts

**DOI:** 10.1080/15476286.2025.2551299

**Published:** 2025-08-22

**Authors:** Yatzu Chiu, Risa Ishida, Yohsuke Moriyama, Jan Grašič, Keiko Kono

**Affiliations:** Okinawa Institute of Science and Technology Graduate University, Okinawa, Japan

**Keywords:** Small non-coding RNAs, cellular senescence, plasma membrane damage, gene regulation, RNA-seq

## Abstract

Cellular senescence is a stable cell cycle arrest associated with upregulated inflammatory responses. Senescent cells contribute to various pathological and physiological processes including organismal ageing and cancer. Cellular senescence can be induced by various cellular stresses including DNA damage, telomere shortening, oncogene activation, and epigenetic alterations. We have shown that plasma membrane damage can also induce cellular senescence. However, common and specific molecular mechanisms among different senescent cell subtypes remain unknown. MicroRNAs (miRNAs) regulate mRNA and rewire gene expression profiles, contributing to multiple processes including cellular senescence. Here, we performed time-resolved miRNA sequencing and compared the results with mRNA sequencing results using cells experiencing plasma membrane damage-dependent senescence (PMD-Sen) and cells undergoing DNA damage response-dependent senescence (DDR-Sen). We found 65 miRNAs that are differentially regulated in PMD-Sen, contributing to 2,495 miRNA-mRNA pairs. Moreover, PMD-Sen and DDR-Sen shared 41 miRNAs across their sets of miRNA-mRNA pairs. Notably, miR-155-5p emerged as the miRNA with the largest number of shared miRNA-mRNA pairs that exhibit a highly negative correlation. These results highlight miR-155-5p as the potential key regulator of PMD-Sen and DDR-Sen.

## Introduction

Cellular senescence, originally described by Hayflick, initially referred to a state of stable growth arrest resulting from the limited proliferative capacity of normal human fibroblasts [1,2]. After serial passaging, the normal human diploid fibroblasts reach replicative exhaustion and stop dividing in vitro. In 1990, Harley and his colleagues demonstrated that this type of senescence, known as replicative senescence (RS), was associate with telomere shortening [3]. Cellular senescence can also be triggered in response to various stressors, including oxidative stress, unresolved DNA damage and oncogene activation [4]. Although different stressors lead to senescence, the DNA damage response (DDR) is commonly involved in these mechanisms [5]. Senescent cells are metabolically active and resistant to apoptotic signals [6–8]. Senescence features are recognized by a combination of phenotypes, including flattened and enlarged cell morphology; increased activity of senescence-associated β-galactosidase (SA-β-gal), the development of a senescence-associated secretory phenotype (SASP), which is the secretome consisting of proinflammatory cytokines, chemokines, and extracellular matrix proteins; and an increase in cell cycle inhibitors such as p53 and p16 [9,10]. Cellular senescence is beneficial to the organism in some contexts including embryonic development, tissue remodelling and tumour suppression. However, cellular senescence can also be deleterious in other contexts including ageing and tumour progression [11].

Recently, we showed that plasma membrane damage-dependent senescence (PMD-Sen) is a novel senescence subtype that is induced by transient plasma membrane damage [12]. The plasma membrane can be damaged and lose its integrity when exposed to stress, such as pore-forming toxins secreted by invading pathogens or during the normal physiological process of muscle contraction [13]. Since plasma membrane damage is ubiquitous in the human body [14], further understanding of the regulatory mechanisms of PMD-Sen would potentially contribute to developing a treatment to extend healthy life span.

Cellular senescence is accompanied by large-scale gene expression changes [15–17]. One of the major regulators underlying such gene expression changes are microRNAs(miRNAs) [18]. miRNAs are small non-coding RNAs consisting of 18–24 nucleotides and were initially discovered in the nematode *C.elegans* by the Ambros and Ruvkun groups in 1993 [19,20]. The majority of miRNA genes are transcribed by RNA polymerase II (Pol II) into primary miRNA transcripts (pri-miRNA) in nucleus. These pri-miRNAs, which can be more than 1 kb, contain a hairpin structure where the miRNA sequences are located. Many pri-miRNAs undergo capping and polyadenylation processes [18,21]. Following transcription, pri-miRNAs are processed by a microprocessor complex, which includes the nuclear RNase III enzyme Drosha and DGCR8, resulting in the formation of stem-loop precursor miRNAs (pre-miRNAs) with a length of approximately 70 nucleotides [22]. The pre-miRNAs are then transported from the nucleus to the cytoplasm by the export receptor Exportin-5 and further processed by an RNase III endonuclease, Dicer [23,24]. In cytoplasm, cleavage to the terminal loop of pre-miRNA by Dicer generates the approximately 22-nucleotide miRNA duplex with 2-nucleotide 3’overhangs. Through interaction of miRNA seed sequences (nucleotide 2–7) with the 3ʹ-untranslated region (3’UTR) of the target mRNAs, miRNAs usually repress gene expression by conducting mRNA degradation and/or inhibiting protein translation. It has been estimated that over 60% of the human protein-coding genes contain at least one conserved miRNA-binding site, indicating that miRNAs serve as a mechanism by which genes with diverse functions can be simultaneously regulated [25]. Therefore, miRNAs are believed to play essential roles in diverse biological processes, including development, differentiation, proliferation, and apoptosis [18,26,27]. The capabilities of miRNAs in controlling various biological processes enable miRNAs to be tools to explore the key pathways that govern specific cell fates [28].

In this study, we focused on miRNA – mRNA interactions as a post-transcriptional regulatory mechanism contributing to senescence. Although the transcriptional and epigenetic regulation of senescence has been extensively investigated [29,30], the role of miRNAs in orchestrating subtype-specific senescence remains insufficiently understood. By integrating time-resolved miRNA and mRNA expression profiling, we sought to identify both shared and subtype-specific miRNA – mRNA regulatory axes. We identified 2,495 negatively correlated miRNA – mRNA pairings involving 65 miRNAs that are regulated in PMD-Sen. Moreover, PMD-Sen and DNA damage response-dependent senescence (DDR-Sen) shared 41 miRNAs across their sets of miRNA-mRNA pairs. Notably, miR-155-5p exhibited the largest number of shared target mRNAs with strong negative correlation, suggesting a central role in regulating core aspects of the senescent phenotype.

## Results

### The induction of plasma membrane damage-dependent senescence and DNA damage response-dependent senescence in WI-38 normal human fibroblasts

**Figure 1.**
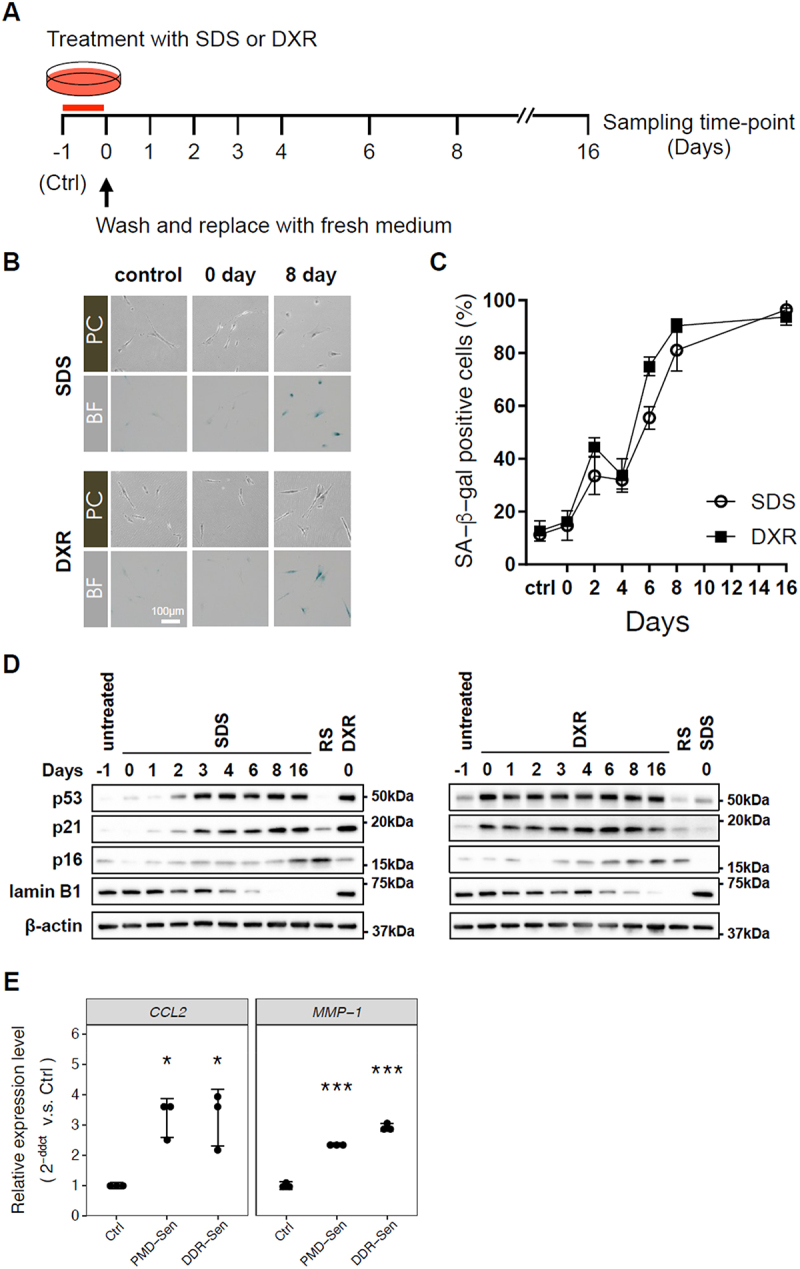
Characterization of plasma membrane damage-dependent senescence (PMD-Sen) and DNA damage-induced senescence (DDR-Sen) in WI-38 cells.

We previously showed that plasma membrane damage can induce cellular senescence (PMD-Sen) in normal human fibroblasts including WI-38 cells and BJ cells in vitro, and performed the time-resolved mRNA seq analysis (GSE222400) [12]. Here, we aim to identify the miRNAs that would underly the mRNA expression changes that were identified in our previous work. WI-38 cells were treated with sodium dodecyl sulphate (SDS)-containing medium for 24 hours, washed away, and incubated with DMEM for additional 16 days. As a control, we utilized DNA damage response-dependent senescent (DDR-Sen) cells that were induced by doxorubicin (DXR) treatment for 24 hours (Figure 1A). Both PMD-Sen cells and DDR-Sen cells displayed the well-established cellular senescence feature of increased SA-β-gal staining positive cells (Figure 1B,C). SA-β-gal staining suggests that senescence progressed over time, with senescent cells dominating the culture by D8. PMD-Sen cells and DDR-Sen cells showed increased senescent cell marker proteins, including p53, p21, and p16, in addition to the decrease of lamin B1 (Figure 1D). Increase in p16 and decrease in lamin B1 were also observed in another senescent cell control, replicative senescence (RS) cells, that were induced by repeated cell division and reached at least population doubling level of 50. The increased levels of p53 and p21 suggest that the senescence process began on D2 in PMD-Sen cells and on D0 in DDR-Sen cells. Moreover, increased mRNA levels of SASP factors, *CCL2* and *MMP1*, were observed (Figure 1E). Collectively, these results confirm successful cellular senescence induction.

(A) The experimental design of time-series mRNA profiling in PMD-Sen and DDR-Sen encompassed eight time points (0, 1, 2, 3, 4, 6, 8, and 16 days following SDS or DXR removal) with duplicate samples. Untreated, proliferating WI-38 cells served as the control group (labelled Ctrl or D-1). (B and C) Visualization and quantitative assessment of SA-β-gal activity in WI-38 cells under various conditions: untreated control (Ctrl), plasma membrane damage-induced senescence (PMD-Sen), and DNA damage-induced senescence (DDR-Sen). Cells were stained at the indicated time points after treatment with either sodium dodecyl sulphate (SDS) or Doxorubicin (DXR) for 24 hours. Scale bar represents 100 μm. (D) Detection of senescence marker proteins (p16, p21, p53, and Lamin B1) in PMD-Sen and DDR-Sen at specified intervals by western blot, with β-actin as the loading control. (E) Transcriptional profiling of senescence-associated secretory phenotype (SASP) genes by RT-qPCR in WI-38 cells exposed to SDS or DXR. * *p* < 0.05, ** *p* < 0.01, *** *p* < 0.001.

### Time-resolved miRNA profiling revealed dynamic changes of differentially expressed miRnas during senescence induction

**Figure 2.**
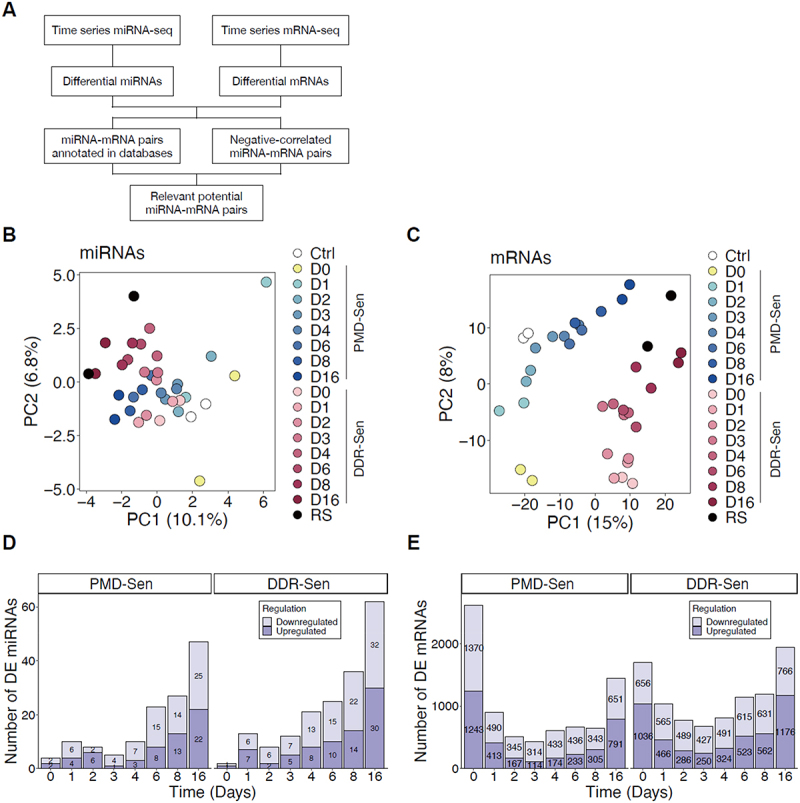
Temporal analysis of transcriptional profiles elucidated the dynamic changes in differentially expressed miRnas and mRNAs following senescence induction.

To investigate the regulatory role of miRNAs in senescence, we conducted time-resolved miRNA sequencing (miRNA-seq) and mRNA sequencing (mRNA-seq) using PMD-Sen cells and DDR-Sen cells. The WI-38 cells were treated with either SDS or DXR for 24 hours, washed away, and incubated for additional 16 days, with samples collected on days 0, 1, 2, 3, 4, 6, 8, and 16 (Figure 1A). These samples were then subjected to miRNA-seq and mRNA-seq to elucidate the temporal dynamics of miRNA and mRNA expression during the senescence process. To identify potential miRNA–mRNA interaction during senescence, we performed an integrative analysis of time-resolved miRNA-seq and mRNA-seq (Figure 2A). Specifically, we focused on negatively correlated miRNA-mRNA pairs because miRNAs regulate gene expression primarily by negatively regulating gene expression. Potential miRNA-mRNA pairs were determined by intersecting the pairs exhibiting inverse expression profile correlations (Spearman’s correlation coefficients < 0.5) with those annotated by multiMiR [31], an R package for miRNA – mRNA interaction analysis that collects 14 different databases such as miRTarBase [32], DIANA-microT [33], miRDB [34], TargetScan [35] and others.

(A) A workflow diagram illustrates the process of identifying miRNA-mRNA pairs through integration of sequencing data and bioinformatic analysis employing the R package multiMiR. (B and C) Two component Principal Component Analysis (PCA) plots display the time-series miRNA (B) and mRNA (C) expression profiles for PMD-Sen, DDR-Sen, and replicative senescence (RS) in WI-38 cells. Time points and senescence subtypes are represented by distinct colours. Untreated proliferating WI-38 cells were utilized as the control (Ctrl). D0: after 24 hours of senescence induction treatment, prior to washout, D1: one day post-washout. (D and E) The total number of differentially expressed miRNAs (D) and mRNAs (E) at each time point for PMD-Sen and DDR-Sen, defined by a P-value < 0.05 and an absolute log2 fold change > 1.

**Figure 3.**
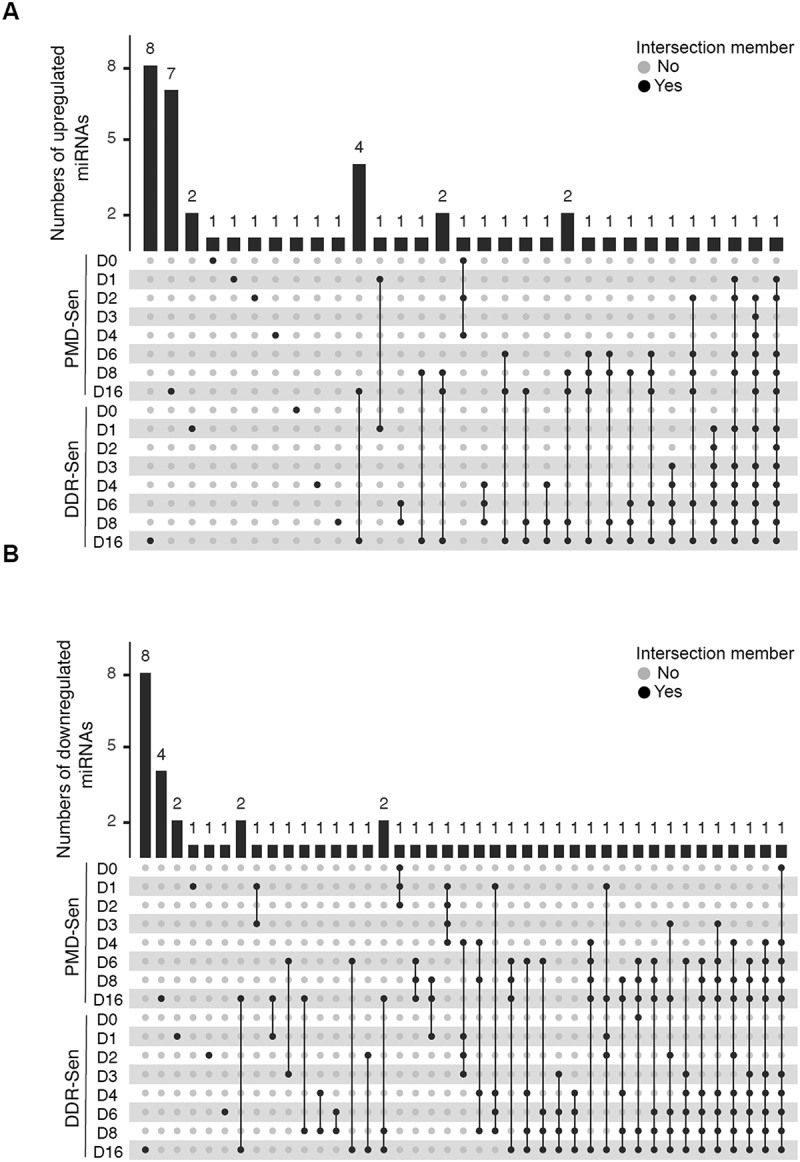
Comparative analysis revealed shared or distinct differentially expressed miRnas in the process of PMD-Sen and DDR-Sen.

First, differential expression analysis of miRNAs and mRNAs between proliferating untreated cells and senescence-induced cells were conducted using DESeq2 [36]. Principal Component Analysis (PCA) based on the log10-transformation of DESeq2-normalized counts in miRNA-seq and mRNA-seq demonstrated the variances of miRNA expression or mRNA expression across the process of senescence induction (Figure 2B,C). The PCA revealed a progressive shift in gene expression over time (from control [Ctrl], day 0 [D0] to day 16 [D16]), supporting a progressive transcriptional shift during senescence. Furthermore, the variance between PMD-Sen and RS or between DDR-Sen and RS decreased along the time course. Notably, both miRNAs and mRNAs of D0 sample in PMD-Sen showed different trends from any other time points, indicating a distinct cellular event at D0 (after 24 hours of SDS treatment, prior to washout) in PMD-Sen. Based on the thresholds of *p*-value < 0.05 and absolute log2 Foldchange > 1, we detected 134 and 179 differentially expressed (DE) miRNAs in PMD-Sen and DDR-Sen, respectively, when compared to the untreated control (Ctrl) (Figure 2D). Analogously, a total of 7822 and 9263 DE mRNAs were identified in PMD-Sen and DDR-Sen, respectively (Figure 2E). To further characterize the unique miRNA profile in different subtypes of senescence, we performed a comparative analysis of the compositions of DE miRNAs among PMD-Sen, DDR-Sen, and RS. The UpSet diagram illustrated the numbers of unique DE miRNAs in each senescence subtype, highlighting senescence subtype-specific DE miRNA expression patterns. For instance, in PMD-Sen, 22 upregulated miRNAs were observed at D16, of which 7 miRNAs were exclusively upregulated at D16 in PMD-Sen rather than at any other time points or in other subtypes (Figure 3A and Supplementary Table S1). Among the downregulated miRNAs in PMD-Sen, 4 of a total 25 downregulated miRNAs at D16 were identified as unique signatures of PMD-Sen_D16 (Figure 3B and Supplementary Table S2). These observations suggest that miRNA profiles may be dependent on the subtypes of senescence. Furthermore, several senescence-associated miRNAs were identified, which verifies our analysis methods (Table 1). For instance, miR-664a-3p [37], miR-486-5p [38], the miR-30-5p family [39], and miR-335-5p [40], which have been previously reported as upregulated in senescence, were also found to be increased in our dataset. Conversely, miR-17-5p [41,42], miR-106b-5p [43], and miR-625-3p [44], which are known to be downregulated in senescence, were also consistently downregulated in our analysis.

UpSet plot illustrating the overlap of differentially expressed miRNAs between SDS and DXR post-treatment days for upregulated (A) or downregulated (B) miRNAs. The isolated circles indicate no overlap with other samples. Linked circles represent the specific intersections of differentially expressed miRNAs. The top bar graph quantifies the differentially expressed miRNAs for each distinct or intersecting group.

**Table 1.** Known senescence-associated miRnas identified in the present study.(chiu et al).

miRNA	Expression Change	Finding in Senescence/Ageing
miR-664a-3p	Commonly upregulated in PMD-Sen_D02, D03, D04, D06, D08, and D16; DDR-Sen_D01, D03, D04, D06, D08, and D16	Upregulated in replicative senescence [35]
miR-486-5p	Commonly upregulated in DDR-Sen_D01, D02, D03, D04, D06, D08, and D16	Upregulated in replicative senescence and etoposide-induced senescence [36]
miR-30-5p family	Commonly upregulated in DDR-Sen_D03, D04, D06, D08, and D16	Upregulated in ionizing radiation-induced senescence [37]
miR-335-5p	Upregulated in DDR-Sen_D16	Upregulated during ageing and prolonged ex vivo expansion [38]
miR-17-5p	Commonly downregulated in PMD-Sen_D04, D08, and D16; DDR-Sen_D02, D04, D06, D08, and D16	Downregulated in hydrogen peroxide-induced senescence and ageing [39,40]
miR-106b-5p	Commonly downregulated in PMD-Sen_D04 and D08; DDR-Sen_D04 and D08	Downregulated during ageing [41]
miR-625-3p	Commonly downregulated in DDR-Sen_D03, D06, D08, and D16	Downregulated in hydrogen peroxide-induced senescence [42]

### Soft clustering and functional enrichment analyses suggested core and senescence subtype-specific biological processes

**Figure 4.**
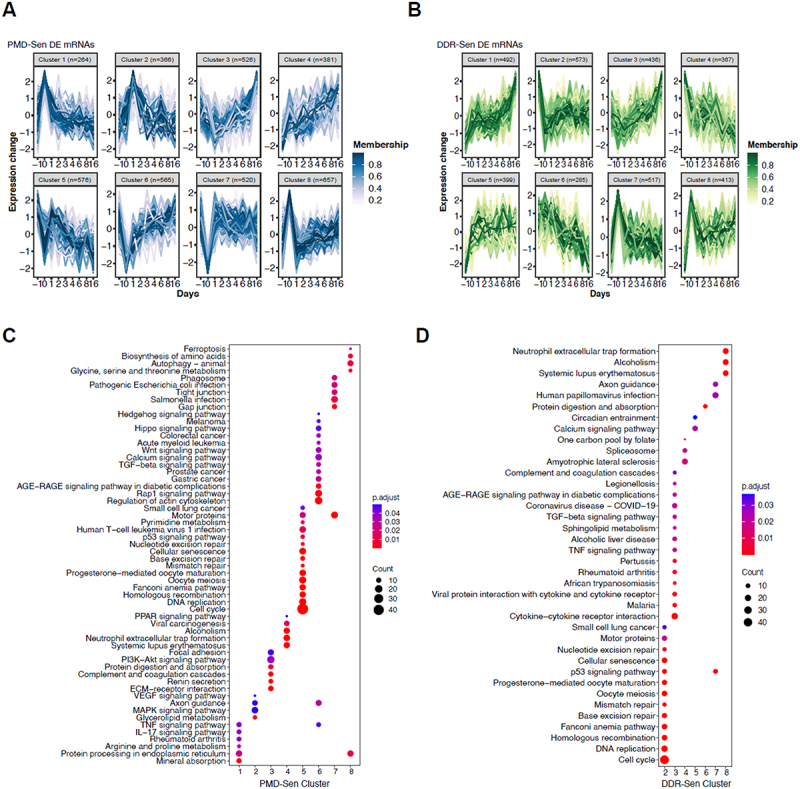
Pathway analysis of the clustered differentially expressed mRNAs during PMD-Sen and DDR-Sen.

To investigate the dynamic changes of DE mRNAs, we implemented soft-clustering analysis of expression profiles (DESeq2-normalized counts) using R package Mfuzz [45]. All DE mRNAs were separated into optimal clusters based on similar temporal expression patterns, which may be involved in common functions during senescence induction. This approach enabled the identification of eight distinct expression pattern clusters in both PMD-Sen and DDR-Sen, each comprising from 264 to 657 DE mRNAs (Figure 4A,B).

(A and B) The R package mfuzz was employed to generate soft clustering profiles for differentially expressed mRNAs in PMD-Sen (A) and DDR-Sen (B). Color intensity correlates with membership strength. (C and D) KEGG pathway enrichment analysis for the clusters of differentially expressed mRNAs from PMD-Sen (C) and DDR-Sen (D). Only the most statistically significant enrichments (Benjamini-Hochberg adjusted p-value < 0.05) are presented.

To gain insight into the biological functions of the genes with distinct patterns during senescence progress, we performed Kyoto Encyclopedia of Genes and Genomes (KEGG) enrichment analysis [46] of DE mRNAs in each cluster utilizing an R package clusterProfiler [47]. We identified key KEGG pathways with a Benjamini-Hochberg adjusted p-value < 0.05, revealing potential biological relevance for each DE mRNA cluster in PMD-Sen and DDR-Sen (Figure 4C,D). In PMD-Sen, Cluster 1 exhibited a highest level at D0, followed by a gradual decline towards D16, and was associated with endoplasmic reticulum protein processing. Cluster 2 peaked at D1 and then decreased, with enrichment in glycerolipid metabolism. Cluster 3 was initially downregulated at D0 and D1 but gradually increased over time, and was associated with ECM–receptor interaction, protein digestion, and the PI3K-Akt signalling pathway. Cluster 4 displayed a rapid upregulation at D0, with a subsequent increase, and was associated with immune system functions and disease-related pathways. Cluster 5 exhibited a sharp downregulation at D0 and the lowest expression at D16, with enrichment in cell cycle regulation, DNA replication, and homologous recombination. Cluster 6 exhibited the lowest expression at D0, followed by a gradual increase, and was enriched in actin cytoskeleton regulation and Rap1 signalling. Cluster 7 showed a downregulation peak at D0, increased at D1, and then slowly declined, primarily associating it with gap and tight junction processes. Finally, Cluster 8 demonstrated an upregulation peak at D0, a decrease at D1, and a modest increase afterwards, which is related to amino acid metabolism and ER protein processing.

In DDR-Sen, Cluster 1 exhibited a gradual increase in expression, reaching its peak at D1, although no significant KEGG pathway enrichment was observed. Cluster 2 experienced a rapid downregulation at D0 and remained at low levels, with strong enrichment in cell cycle regulation, DNA replication, and homologous recombination. Cluster 3 showed a decrease at D0 and D1, followed by an increase over time, similar to PMD-Sen Cluster 3, and was associated with cytokine – cytokine receptor interaction. Cluster 4 demonstrated a continuous decline and was linked to transcriptional regulation, metabolism, and disease-related pathways. Cluster 5 was upregulated at D0 and maintained a high expression, with enrichment in calcium signalling. Cluster 6 declined from D1 and was involved in protein digestion and metabolic pathways. Cluster 7 peaked at D0 and gradually decreased, resembling PMD-Sen Cluster 1, and was significantly enriched in the p53 signalling pathway. Finally, Cluster 8 peaked at D0 and maintained high expression levels, with enrichment in immune response and disease-related pathways.

The soft clustering analysis identified distinct temporal expression patterns, while the KEGG pathway enrichment analysis further suggested that genes within the same cluster may participate in related biological processes during the progression of senescence. Additionally, the KEGG pathway enrichment analysis suggests that different senescent cell subtypes utilize distinct regulatory pathways. Despite these variations, the core pathways of senescence, including the p53 signalling pathway, cellular senescence, and DNA repair mechanisms, were consistent across senescent cell subtypes. Collectively, these findings underscore the distinct yet interconnected molecular pathways involved in PMD-Sen and DDR-Sen.

### miRnas mediate the temporal changes of mRNAs in PMD-Sen and DDR-Sen

**Figure 5.**
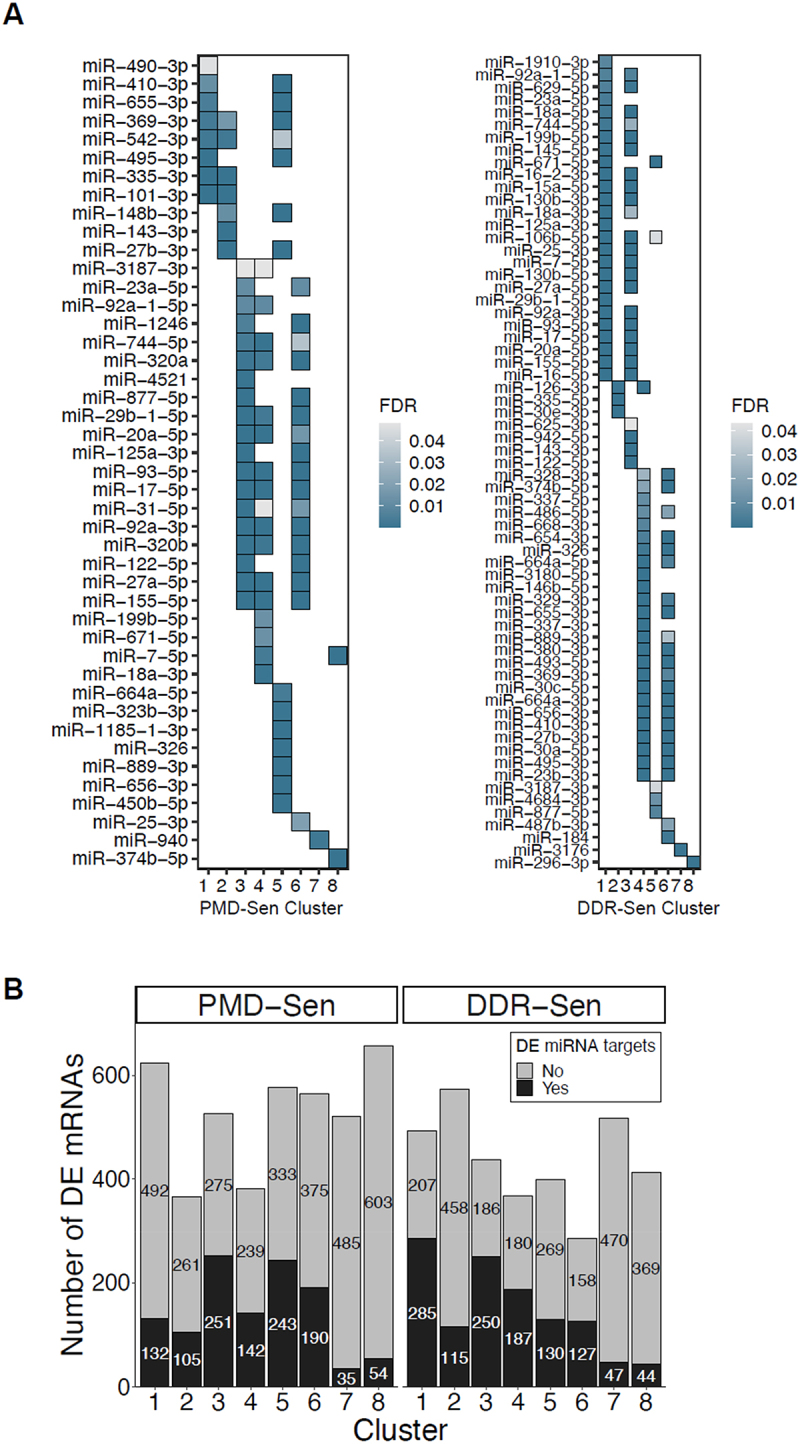
Differentially expressed miRNA target genes are highly enriched among clusters in PMD-Sen and DDR-Sen.

We then sought to quantitively assess the impact of miRNAs on the temporal changes of mRNAs by conducting hypergeometric tests adopting R package hypeR [48]. The results revealed that the targets of DE miRNAs were significantly represented in the clusters of PMD-Sen and DDR-Sen (Figure 5A, Supplementary Fig. S1 and S2). For example, in PMD-Sen, the genes in Cluster 3, 4, 5 and 6 were targeted by a minimum of 14 miRNAs. In PMD-Sen_Cluster 7, there was an enrichment of targets specifically from miR-940. In the case of DDR-Sen, Clusters 1, 3, 4, and 5 individually showed enrichment for targets of at least 25 miRNAs. Furthermore, miR-3176 and miR-296-3p had their targets specifically enriched in DDR-Sen_Cluster 7 and DDR-Sen_Cluster 8, respectively. Collectively, a substantial proportion of the DE mRNAs within the PMD-Sen and DDR-Sen clusters, ranging from 7% to 48%, were targeted by DE miRNAs (Figure 5B). This observation suggests that miRNAs may have important roles in the temporal changes of genes during PMD-Sen and DDR-Sen processes.

(A) Hypergeometric tests were performed using the R package hypeR to evaluate the enrichment of miRNA targets among genes in each cluster. The left and right panels illustrate the results for PMD-Sen and DDR-Sen, respectively. Colour gradients represent false discovery rate (FDR) values. (B) A histogram illustrates the distribution of mRNAs targeted by miRNAs across clusters in PMD-Sen and DDR-Sen. The numbers within each bar correspond to the quantity of mRNAs in the respective clusters.

### miR-155-5p may serve as a universal regulator in PMD-Sen and DDR-Sen via potentially mediating its target mRNAs

**Figure 6.**
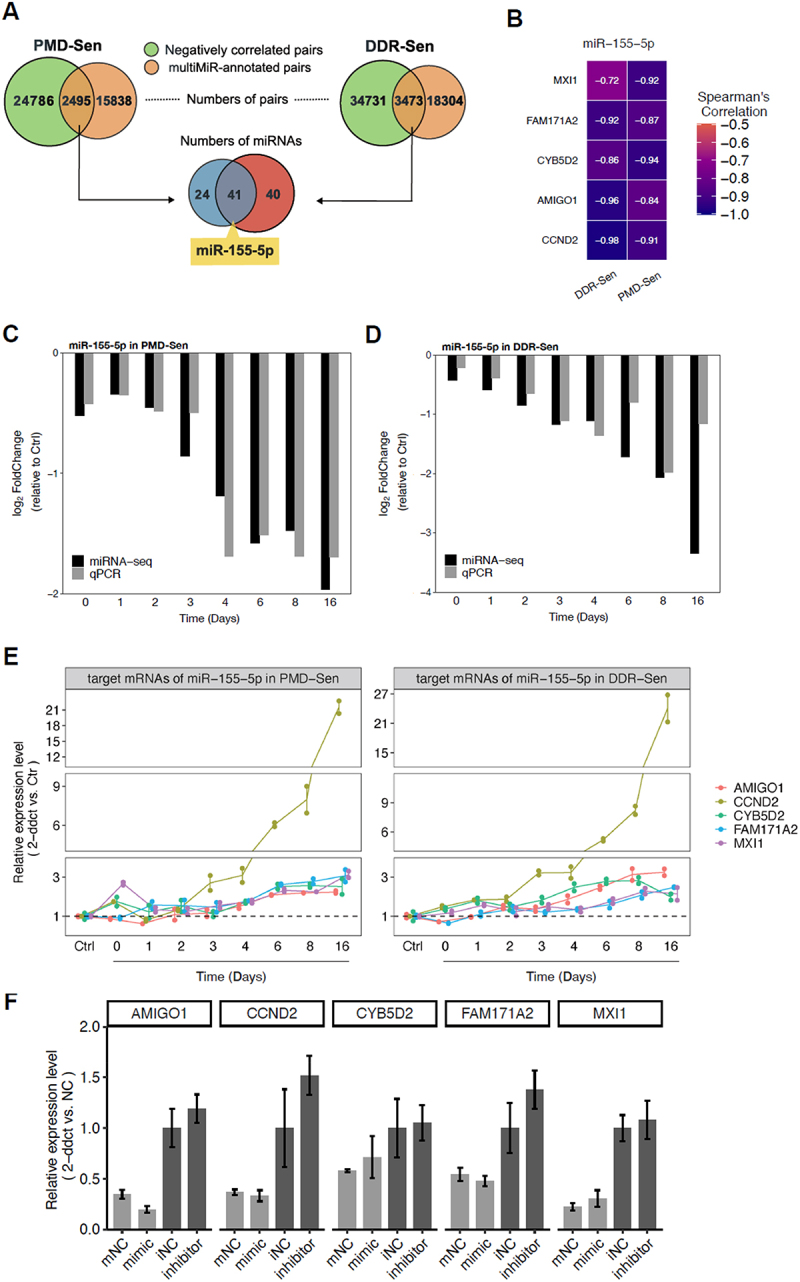
miR-155-5p was identified as a putative regulatory miRNA in both PMD-Sen and DDR-Sen.

To identify the potential regulatory miRNAs that are common in PMD-Sen and DDR-Sen, we compared the miRNA-mRNA interactions identified in PMD-Sen and DDR-Sen. The DE miRNAs and mRNAs were utilized to construct potential miRNA-mRNA pairs by intersecting the negatively correlated pairs and the multiMiR-annotated pairs, as described above. The analysis revealed 27,281 and 38,204 negatively correlated miRNA-mRNA pairs in PMD-Sen and DDR-Sen, respectively (Figure 6A). Among the negatively correlated miRNA-mRNA pairs, 2495 pairs comprising 65 DE miRNAs in PMD-Sen were annotated in multiMiR. In the case of DDR-Sen, 3,473 pairs involving 81 DE miRNAs were identified. These findings suggest that a relatively small number of DE miRNAs potentially associate with a substantial number of DE mRNAs in PMD-Sen and DDR-Sen.

(A) A Venn diagram illustrating the numbers of potential miRNA-mRNA pairings (top panel) and the numbers of miRNAs implicated in these pairings (bottom panel) for PMD-Sen and DDR-Sen. Potential miRNA-mRNA pairs were identified by integrating the negatively correlated pairs obtained through sequencing with the pairs annotated in the multiMiR database, as previously described. (B) Spearman correlation analysis between expression levels of miR-155-5p and its target mRNAs. Only the common pairs exhibiting significant negative correlations (Spearman’s correlation coefficients < 0.7) in both PMD-Sen and DDR-Sen are presented. (C and D) Relative expression of miR-155-5p by RT-qPCR in PMD-Sen (C) and DDR-Sen (D). U6 was used for normalization. (E) RT-qPCR validation of mRNA targets in PMD-Sen (left panel) and DDR-Sen (right panel). (F) Relative expression of mRNA targets by RT-qPCR in WI-38 cells treated with negative control for miRNA mimic (mNC), miRNA mimics, negative control for miRNA inhibitor (iNC), or miRNA inhibitors. GAPDH mRNA was utilized for normalization. Bars represent the means and standard errors of four biological replicates.

Further comparative analysis identified 41 DE miRNAs common to PMD-Sen and DDR-Sen, suggesting their potential universal role in the senescence process across different subtypes (Figure 6A). Of particular interest was miR-155-5p, which had a significant differential expression and formed the greatest number of miRNA-mRNA pairs with strong negative correlations (Spearman’s correlation coefficients < 0.7). Next, a subsequent selection process was conducted to explore the relevant functional miRNA-mRNA pairs implicated in senescence. This process involved the selection of miRNA-mRNA pairs that were either experimentally confirmed or predicted by at least two independent databases [31]. Consequently, the identified targets exhibiting a strong negative correlation with miR-155-5p include *AMIGO1*, *CCND2*, *CYB5D2*, *FAM171A2*, and *MXI1* (Figure 6B).

The expression level of miR-155-5p was further validated by real-time qPCR, showing a similar pattern and strong correlation with the expression level detected by miRNA seq (Figure 6C,D). Moreover, the decrease of miR-155-5p was observed in another human normal fibroblast strain BJ cells of DDR-Sen (Supplementary Fig. S4A-C). These results suggested a reliable inference of miRNA expression signatures through miRNA-seq and bioinformatics analysis, enabling further investigation of the regulatory functions of miR-155-5p involved in PMD-Sen and DDR-Sen. Next, the expression levels of target mRNAs were also validated using real-time qPCR. The results showed an upregulation of these target mRNAs over time (Figure 6E), aligning with the bioinformatics analysis of mRNA sequencing and demonstrating an inverse relationship with the downregulated miR-155-5p, as expected.

To further investigate the influence of miR-155-5p on its target mRNAs, transient transfection was performed with miRNA mimics (small RNA duplexes imitating mature miRNA) and inhibitors (antisense RNA oligos that bind to and inhibit miRNA) to induce miRNA overexpression and inhibition, respectively. The experimental results revealed that the expression of miR-155-5p target mRNAs (*AMIGO1*, *CCND2*, *CYB5D2*, *FAM171A2*, and *MXI1*) exhibited downregulation in response to the miR-155-5p mimic and upregulation when exposed to the miR-155-5p inhibitor; however, these changes did not reach statistical significance (Figure 6F). These results suggest a potential regulation of these mRNAs by miR-155-5p, which needs further validation.

## DISCUSSION

miRNAs play essential roles in diverse biological processes, including development, differentiation, proliferation, and apoptosis [26,38]. Furthermore, several miRNAs have been reported as senescence-associated miRNAs [49]. In this study, we performed time-resolved miRNA profiling in PMD-Sen, a recently discovered senescent cell subtype, utilizing human fibroblast WI-38 cells. To establish the interactome of miRNAs and their target mRNAs in PMD-Sen, an integrative analysis was performed based on miRNA and mRNA expression profiles and miRNA-mRNA databases (Figure 2B). This approach aimed to shed light on the potential regulatory mechanisms by which miRNAs modulate the expression of mRNAs implicated in PMD-Sen. Although a limited number of cases demonstrating positive correlation patterns between expression levels of miRNAs and mRNA targets have been reported [50], miRNA target prediction is frequently supported by utilizing the inverse correlation between miRNAs and their target mRNAs [18,51]. In our analysis, we used miRNA-mRNA pairs exhibiting negative correlation as the principal components in the construction of regulatory networks.

In the PCA analysis of the time-series miRNA profiles, we observed that the miRNA expression profile at D0 (after 24 hours of senescence induction treatment, prior to washout) had the least similarity to other time points, and the miRNA signatures subsequently progressed towards replicative senescence. This pattern suggests that the differentially expressed miRNAs at D0 might be implicated in the initial response to plasma membrane injury, rather than in the induction of PMD-Sen. Furthermore, the miRNAs expressed from D1 (1-day post-washout) through D16 (16 days post-washout) potentially function as regulators of the induction and maintenance of PMD-Sen. Additionally, an increase in the number of differentially expressed miRNAs was observed over time following plasma membrane damage, suggesting a temporal correlation between these dysregulated miRNAs and the progression of PMD-Sen. Comparative analyses between PMD-Sen, DNA damage response-dependent senescence (DDR-Sen), and replicative senescence (RS) revealed both shared and unique miRNAs, supporting the idea that senescence subtype – specific stressors induce distinct regulatory mechanism (Supplementary Figure S3; Supplementary Table S6 and S7). For example, while DDR-Sen and RS rely on DNA damage response signalling, PMD-Sen does not exhibit detectable DNA damage at early stages [12,52]. This distinction is reflected in the differential miRNA signatures. miR-1260b was uniquely upregulated at D0 in PMD-Sen, but not at later stages or in DDR-Sen or RS, suggesting its potential role in the acute response to membrane damage. In contrast, miRNAs such as miR-27b-3p, miR-146b-5p, and miR-126-3p were upregulated at PMD-Sen-D16, DDR-sen-D16 and replicative senescence, suggesting their involvement in general mechanisms of senescence. In a related study, Wang et al. [53] identified eight miRNAs that were differentially expressed across senescent cell subtypes: replicative, ionizing radiation-induced, and busulfan-induced senescence. Furthermore, they observed at least 20 miRNAs that were uniquely differentially expressed in each senescent cell subtype. Consistent with their findings, our comparative analysis suggests that distinct miRNA dysregulation patterns may vary depending on the trigger of cellular senescence, while certain miRNAs potentially function as universal regulators of multiple senescent cell subtypes.

Soft clustering of temporal mRNA expression profiles unveiled the dynamic nature of gene expression patterns in PMD-Sen and DDR-Sen. Notably, a substantial proportion of these genes were identified as putative targets of the differentially expressed miRNAs, indicating a potential involvement of miRNA-mediated regulation in the mechanisms underlying senescence. Through the construction of miRNA-mRNA networks and subsequent functional annotation, we identified the miRNAs that potentially play crucial roles in the response to plasma membrane damage and the mechanism underlying PMD-Sen. For instance, the initial transient upregulation of genes at D0 in PMD-Sen_Cluster 1, governed by miRNAs such as miR-495-3p, which was previously linked to protein folding [54], suggests an adaptive mechanism involving endoplasmic reticulum protein processing immediately after plasma membrane damage. Notably, *DDIT3*, *HERPUD1*, and *HSPA6* were among the most strongly upregulated genes (log₂FC > 3, adjusted *p* < 0.05). The rapid, transient upregulation of genes, such as *IL1B*, *AREG*, and *CSF1R*, at D1 in PMD-Sen_Cluster 2, primarily targeted by miRNAs like miR-101-3p, miR-143-3p, and miR-148-3p, highlights their potential role in PMD-Sen through modulation of the MAPK signalling pathway, a well-established regulator of cellular senescence [55–57]. PMD-Sen_Cluster 3 was initially downregulated and gradually increased, and was association with the PI3K-Akt signalling pathway. Previous study by Astle et al. [58] demonstrated that activated PI3K-Akt induced senescence independently of DNA damage, consistent with our previous observation of the absence of detectable DNA damage during early PMD-Sen [12]. PMD-Sen_Cluster 4 exhibited a rapid (D0) and sustained upregulation, associated with PPAR signalling pathways, which plays a crucial role in regulating lipid metabolism and promoting cell survival [59]. PMD-Sen_Cluster 5 was enriched in cell cycle and DNA replication, indicating roles in senescence establishment and/or maintenance. It is noteworthy that the genes in DDR-Sen_Cluster 2 also showed significant enrichment in cell cycle and DNA replication pathways, and predominantly comprised genes that were putative targets of 3 miRNAs (miR-126-3p, miR-30e-3p, and miR-35-5p), which are different from PMD-Sen. These observations suggest the presence of distinct miRNAs functioning in cell cycle and DNA replication processes across PMD-Sen and DDR-Sen pathways. PMD-Sen_Cluster 6 revealed the lowest levels at D0, followed by a gradual upregulation from D1 to D16, and was enriched in actin cytoskeleton and Rap1 signalling, suggesting involvement in cell adhesion and motility [60,61]. PMD-Sen_Cluster 7 demonstrated a significant reduction in gene expression at D0, which was followed by an increase at D1 and a gradual decline thereafter. This cluster was primarily linked to gap and tight junction pathways. In contrast, Cluster 8 reached its highest expression at D0, decreased at D1, and subsequently returned to levels akin to those of untreated cells (D-1). The genes in this cluster were identified as predicted targets of miR-374b-5p and miR-7-5p. KEGG analysis revealed an enrichment in pathways related to amino acid biosynthesis and metabolism, which are crucial for protein synthesis [62]. These observations suggest that miR-374b-5p and miR-7-5p may contribute to the regulation of early protein synthesis in response to plasma membrane damage.

Of particular interest, miR-155-5p emerged as a potential common regulator impacting multiple clusters across both PMD-Sen (Clusters 3, 4, 6) and DDR-Sen (Clusters 1, 3), and its expression was downregulated in all senescence models including RS. Its predicted targets – such as *CCND2*, *MXI1*, and *CYB5D2*—were upregulated in all three subtypes and are known to participate in cell cycle arrest, stress responses, and senescence-associated signalling, suggesting the involvement of miR-155-5p in senescence regulation. The upregulation of early senescence markers, p53 and p21 occurs prior to the decrease in miR-155-5p (Figures 1B-D and 6C,D). These results support the interpretation that the decrease in miR-155-5p most likely contributes to the maintenance, but not the induction, of senescence. In BJ cells (another normal human fibroblast), we found that the increase in SA-β-gal staining positive cells was slower in PMD-Sen cells than in DDR-Sen cells, and miR-155-5p decreased on D8 in DDR-Sen cells but not in PMD-Sen cells (Supplementary Fig S4A-C). These results are consistent with the idea that the downregulation of miR-155-5p occurs during late senescence.

In addition to the miRNAs predicted to regulate temporally clustered mRNAs, identified through soft clustering, we conducted a separate analysis of KEGG pathway enrichment using subtype-specific miRNA – mRNA pairs, regardless of whether the mRNA targets were included in the clustering (Supplementary Table S4 and S5). This analysis aimed to elucidate broader subtype-specific regulatory programs. PMD-Sen-specific miRNA – mRNA pairs exhibited enrichment in pathways such as FoxO signalling, PI3K-Akt signalling, and TNF signalling, which are implicated in cell survival, oxidative stress responses, and inflammation – characteristics of non-genotoxic stress adaptation. In contrast, DDR-Sen-specific pairs were enriched in the p53 signalling pathway, Fanconi anaemia pathway, and cellular senescence, aligning with canonical DNA damage responses and checkpoint activation. Moreover, shared miRNA – mRNA pairs between PMD-Sen and DDR-Sen were enriched for the AGE-RAGE signalling pathway, which integrates inflammation, oxidative stress, and cell survival. miR-155-5p emerged as a central node in this network, but other shared miRNAs (e.g. miR-126-3p, miR-369-3p, miR-495-3p) also targeted genes within the pathway, highlighting a cooperative regulatory network within the pathway associated with cell fate determination. Collectively, these findings complement the soft clustering-based analysis and highlight how both dynamic and subtype-specific regulatory axes contribute to senescence.

We recognize several limitations in this study. First, the use of only two biological replicates per time point for miRNA-seq and mRNA-seq. While we applied multiple layers of analysis to strengthen the findings, future studies with experimental validation will be essential to confirm these observations. Second, while we identified potential regulatory interactions, such as miR-155-5p targeting *CCND2*, *MXI1*, and *CYB5D2*, direct physical interactions were not assessed. Third, the consequences of miR-155-5p downregulation should be further investigated by assessing SASP expression and SA-β-gal activity. These validations will be important for confirming the underlying mechanisms in future work.

Overall, the comprehensive analysis of time-series miRNA-seq and mRNA-seq data elucidates miRNA-mRNA interaction and uncovers miRNA-mediated mechanisms that appear to play a significant role in PMD-Sen progression. Considering the influence of senescent cells on ageing and age-related diseases [11,63], identifying miRNAs that potentially serve as key regulators of PMD-Sen may lead to the development of novel therapeutic strategies utilizing these molecules.

## Materials and methods

### Cell culture and senescence induction

WI-38 human diploid fibroblasts were cultured in Dulbecco’s modified Eagle’s medium (DMEM, FUJI film wako) supplemented with 10% heat-inactivated foetal bovine serum (FBS), 1% penicillin-streptomycin (10,000 U/mL, Gibco) and 1% L-Alanyl-L-Glutamine (200 mmol/L, FUJI film wako). The population doubling level (PDL) was calculated using the formula PDL = PDL_0_ +3.322log(C_f_/C_i_), where C_i_ is the initial cell numbers at seed and C_f_ is the final number of cells at passage.

For PMD-Sen, proliferating WI-38 cells were treated with 0.0095% sodium dodecyl sulphate (SDS) for 24 hours, washed twice, and incubated in DMEM for additional 16 days with medium changes every 2–3 days. For DDR-Sen, proliferating WI-38 cells were treated with 250 nM Doxorubicin for 24 hours, washed twice, and incubated as for PMD-Sen cells. The cells were harvested at 0,1, 2, 3, 4, 6, 8 and 16 days [D0 to D16] after SDS or DXR washout. For replicative senescence (RS), cells were subjected to serial passage until PDL54–56. The proliferating untreated WI-38 cells were used as control.

### Senescence-associated β-galactosidase (SA-β-gal) activity assay

Senescence-associated beta-galactosidase (SA-β-gal) activity was assessed by staining cells with X-gal at pH 6.0 according to Debacq-Chainiaux *et al*. [64]. Briefly, cells were washed with PBS, fixed with 2% paraformaldehyde and 0.2% glutaraldehyde in PBS for 5 minutes, and washed twice with PBS. They were incubated overnight (12–16 hours) at 37°C with beta-galactosidase substrate staining solution containing 40 mM citric acid/Na phosphate buffer (pH 6.0), 5 mM potassium ferricyanide, 5 mM potassium ferrocyanide, 150 mM sodium chloride, 2 mM magnesium chloride and 1 mg/ml X-gal. The cells were washed with PBS twice and observed by standard light microscopy.

### Western blot analysis

Total protein lysates were prepared with 2x Laemmli sampling buffer (4% SDS, 20% glycerol, 2% 2-mercaptoethanol, 0.02% bromphenol blue and 0.125 M Tris HCl). Protein concentrations were determined using Qubit Protein Assay Kit (Invitrogen). Total protein in 2x Laemmli sampling buffer were separated by polyacrylamide gel electrophoresis and then transferred onto PVDF membranes with Trans-Blot Turbo system (Bio-Rad). After blocking with Bullet Blocking One (Nacalai Tesque), the membranes were probed with primary antibodies for p53 (Santa Cruz Biotechnology), p21, p16, or β-actin (Abcam). The horseradish peroxidase-conjugated (HRP) secondary antibody (Cytiva or Cell Signalling) were used for detecting the primary antibodies. Signals were generated by enhanced chemiluminescence and recorded with a ChemiDoc system (Bio-Rad). The densitometric analysis was performed using Image Lab (Bio-Rad).

### RNA extraction and quality control

Total RNA was extracted from cells using TRIzol (Thermo Fisher Scientific) and the RNA Clean & Concentrator kits or Direct-zol Mini Kit (Zymo) according to the manufacturers’ instructions including the digestion with DNase I. The RNA quantity and purity were analysed by NanoDrop spectrophotometer (Thermo Fisher Scientific) and 4200 TapeStation system (Agilent) with RIN number > 9.1.

### RT-qPCR analysis

Total RNA was reverse-transcribed (RT) using SuperScript IV VILO master mix (Thermo Fisher Scientific) according to the manufacturers’ protocols. Briefly, cDNA was synthesized at 25°C for 10 minutes, 50°C for 10 minutes and 85°C for 5 minutes. The PowerTrack SYBR Green master mix (Thermo Fisher Scientific) and gene-specific primers were then applied to perform real-time quantitative (q) PCR analysis on a QuantStudio Real-Time PCR system (Thermo Fisher Scientific) at 95°C for 2 minutes, followed by 40 cycles of 95°C for 5 seconds and 60°C for 30 seconds. Forward and reverse primers are listed in Supplementary Table S2. The relative fold changes of gene expression were calculated by the 2^−ΔΔCt^ method [65] and normalized to GAPDH mRNA.

For the analysis of miRNA, total RNA was processed using the TaqMan MicroRNA Reverse Transcription Kit with the RT primers provided with the TaqMan™ Advanced miRNA Assays (Thermo Fisher Scientific), and the converted cDNA was used as a template for qPCR using TaqMan Advanced miRNA Assays and TaqMan Fast Advanced Master Mix (Thermo Fisher Scientific). Reactions were performed according to the manufacturer’s instructions. U6 snRNA was used as the endogenous control for normalization.

### mRNA sequencing (mRNA-seq) and bioinformatics analysis

The RNA samples were converted into libraries using NEBNext Ultra^TM^ II Directional RNA Library Prep Kit (NEW ENGLAND BioLabs Inc.) according to the manufacturer’s protocol. The high-quality libraries were guaranteed with more than 92% of bases of Q-score > 30. Next-generation sequencing was then carried out on the NovaSeq 6000 System (Illumina) with paired-end sequencing. This work was performed at the Sequencing Section of Okinawa Institute of Science and Technology Graduate University.

To analyse the result of next-generation sequencing dataset, bioinformatic analysis was performed in Python (version 3.7.0) and R (version 4.2.0). Briefly, read quality control and adapter trimming was carried out with Trim Galore (version 0.6.4). The cleaned sequencing reads were processed using HISAT2 (version 2.2.0) and featureCounts (version 2.0.1) for alignment and quantification. The human genome of reference GRCh38 was used. The differential expression analysis was performed using DESeq2 (version 1.36.0). The Wald test was used to determine statistical significance. Differentially expressed mRNAs were defined with P-value less than 0.05 and absolute log2 fold change greater than 1.

### Time-series clustering of differentially expressed genes

The time series significantly differentially expressed genes (P-value less than 0.05 and absolute log2 fold change greater than 1) obtained from mRNA-seq were clustered using Mfuzz package (version 2.56.0) in R based on the fuzzy c-means algorithm. Average DESeq2-normalized counts (replicates at each time point) of individual genes were used as input values for soft clustering.

Pathway annotation of Kyoto Encyclopedia of Genes and Genomes (KEGG) pathway of the gene clusters was conducted using clusterProfiler package (version 4.4.4) in R. Identified KEGG pathways with corrected P-values (Benjamini-Hochberg method) less than 0.05 were considered to be meaningful enrichments.

### miRNA sequencing (miRNA-seq) and bioinformatics analysis

The sequencing libraries were generated using NEXTFLEX Small RNA-Seq Kit v3 (PerkinElmer) following the manufacturer’s protocol from 100 ng of total RNA. Briefly, RNA samples were ligated with 5’ and 3’ adapters, followed by reverse transcription and PCR. PCR amplifications were performed with 17 cycles. The size selection was performed using BluePippin (Sage Science) with a range of 135–165 bp. Finally, libraries were sequenced on Illumina MiSeq system. The high-quality libraries were guaranteed with more than 97% of bases of Q-score > 30. This work was performed at the Sequencing Section of Okinawa Institute of Science and Technology Graduate University.

The raw reads of miRNA-seq were processed using the exceRpt small RNA-sequencing Pipeline (version 4.6.2) available on Genboree (http://www.genboree.org/site/, developed by the Data Integration and Analysis Component of the Extracellular RNA Communication Consortium) for trimming, alignment and counting. Trimming was performed following the manufacturer’s instruction (NEXTFLEX ® Small RNA-Seq Kit v3). The clean reads were mapped to the human genome GRCh38 and aligned against miRbase to create miRNA counts. Differential expression analysis of the gene counts was performed in R (version 4.2.0) using the DESeq2 package (version 1.36.0). The Wald test was used to determine statistical significance. Differentially expressed miRNAs were defined with P-value less than 0.05 and absolute log2 fold change greater than 1.

Predicted miRNA-target interactions were determined by utilizing the multiMiR package (version 1.18.0) in R, which covered 14 publicly available databases including miRecords, miRTarBase, TarBase, DIANA-microT, ElMMo, MicroCosm, miRanda, miRDB, PicTar, PITA, TargetScan, miR2Disease, PharmacomiR and PhenomiR. The miRNA-target interactions were collected with the criterion of top 10% predicted target sites of each external database.

### Identification of the miRnas as key regulators for differentially expressed genes in each cluster

Considering the inverse correlated relationship between miRNAs and mRNAs, Spearman’s correlation coefficients between expression levels of the differentially expressed miRNAs and the differentially expressed mRNAs were calculated. Next, the miRNA-mRNA pairs with Spearman’s correlation coefficients less than −0.5 were intersected with the predicted pairs detected by multiMiR. The negatively correlated predicted pairs were then chosen as putative miRNA-mRNA interactions in the current study. Finally, an enrichment analysis with hypergeometric tests was adopted to quantitatively assess the effects of the differentially expressed miRNAs on the differentially expressed mRNAs in each cluster using hypeR package (version 1.12.0) in R.

### Transfection of miRnas mimics or inhibitors

Cells were seeded 24 hours before transfection to reach a confluency of approximately 70–80%. Transfection was performed using 10 nM of either mirVana miRNA mimics or inhibitors (Thermo Fisher Scientific) and Lipofectamine RNAiMAX Transfection Reagent (Thermo Fisher Scientific), following the manufacturer’s protocol. Briefly, RNAiMAX and the miRNA mimic/inhibitor were mixed and incubated at room temperature for 5 minutes to allow formation of lipoplexes. The complexes were then added dropwise to the cells. After 24 hours of incubation under standard culture conditions, cells were harvested for total RNA extraction and RT-qPCR analysis. All transfections were performed in triplicate.

### Statistical analysis

Data are presented as the means ± standard error of the mean. For comparisons between two groups, Student’s t-test was used. For comparisons involving more than two groups, one-way ANOVA followed by Dunnett’s post-hoc test was performed. *p* < 0.05 was considered as statistical significance.

## Supplementary Material

Supplemental Material

## Data Availability

The miRNA-seq data were deposited in the Gene Expression Omnibus repository of the NCBI database under GEO Series accession number GSE299871; RNA-seq data from previous study are available under GEO Series accession number GSE222400 (the token: ilipuaagzlqjdkb). These data will be publicly available after acceptance of this manuscript. Code for expression analysis is available at https://github.com/YatzuC/small_RNAseq.git. Table S1-S7 are available via figshare. https://figshare.com/s/51ba750b4314c4cc1edc.
