# Supplementary figures and images for: Time-resolved miRNA-mRNA integrated analysis reveals the miRNA-mRNA networks underlying plasma membrane damage-dependent senescence and DNA damage response-dependent senescence in WI-38 normal human fibroblasts
Source: RNA Biol. 2025 Aug 22;22(1):1–19. doi: 10.1080/15476286.2025.2551299 (PMC12407646; doi:10.1080/15476286.2025.2551299)

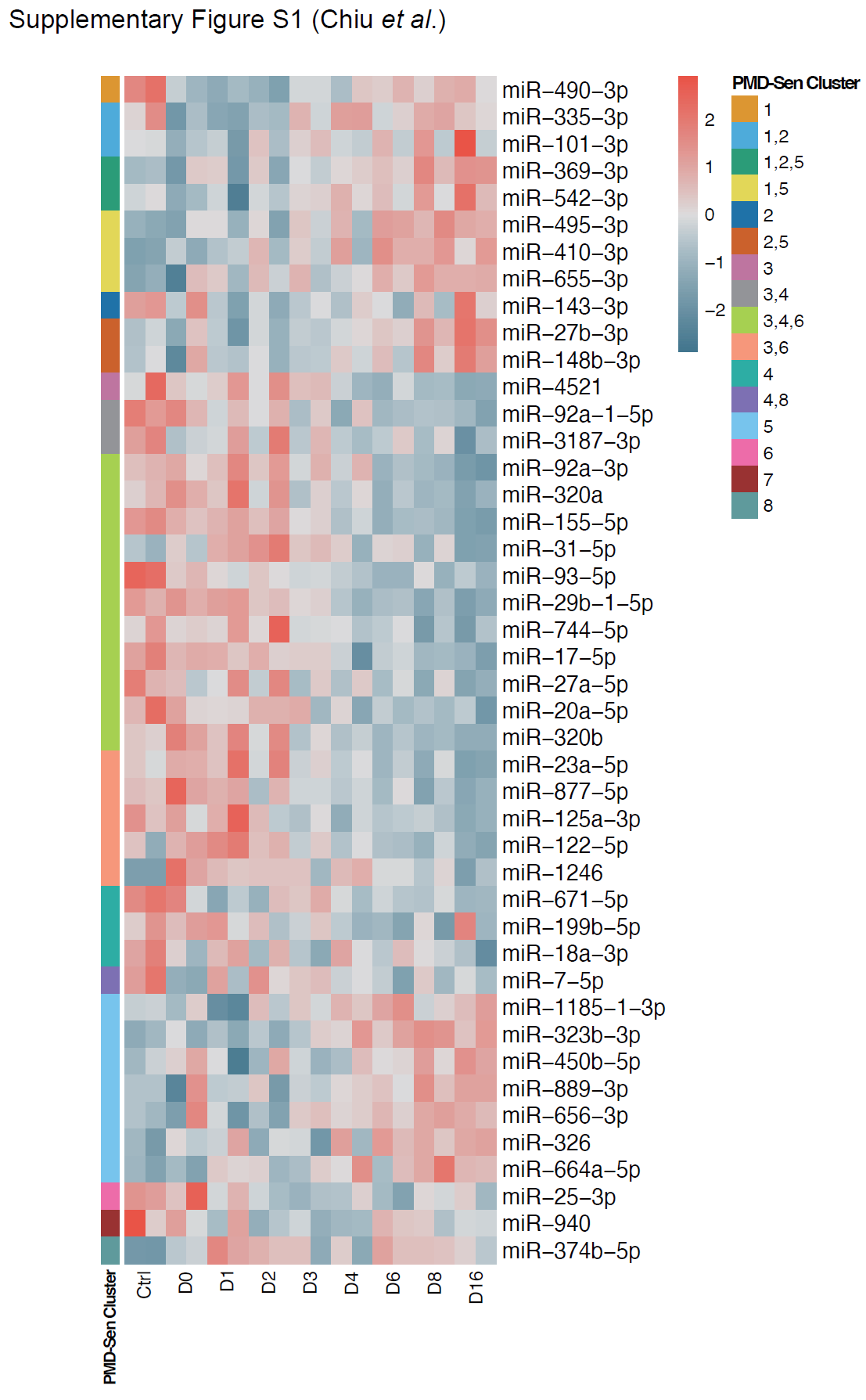


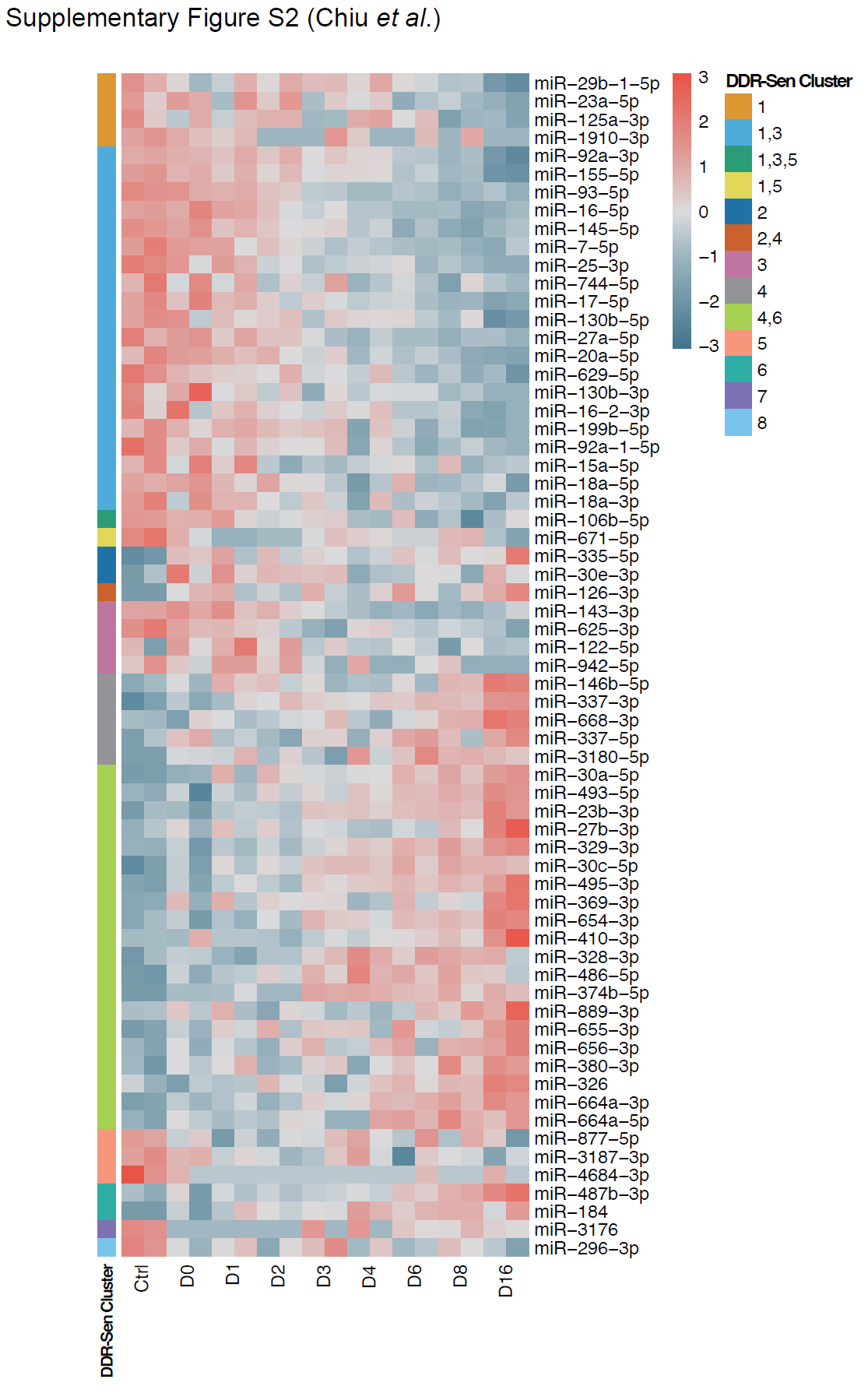


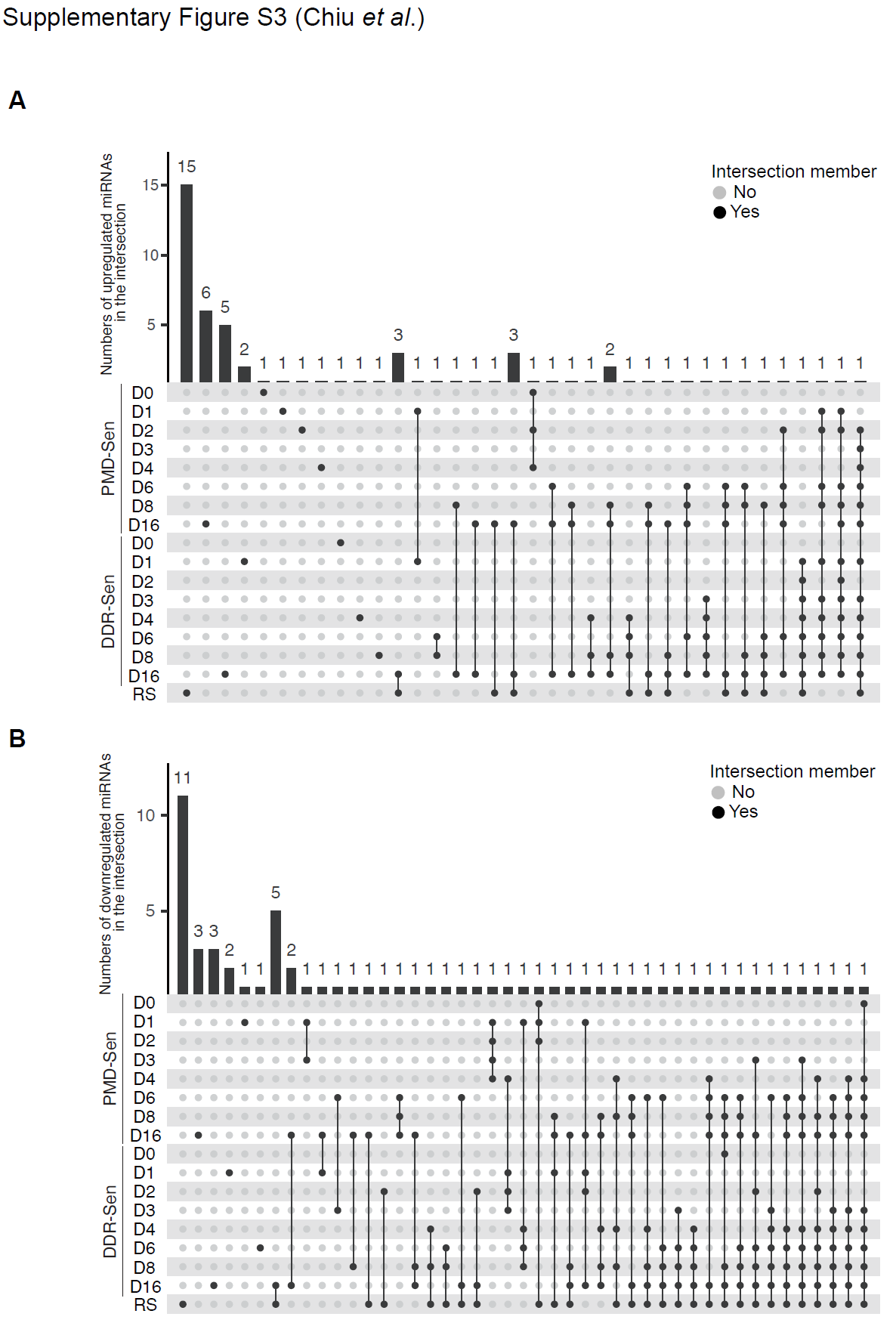


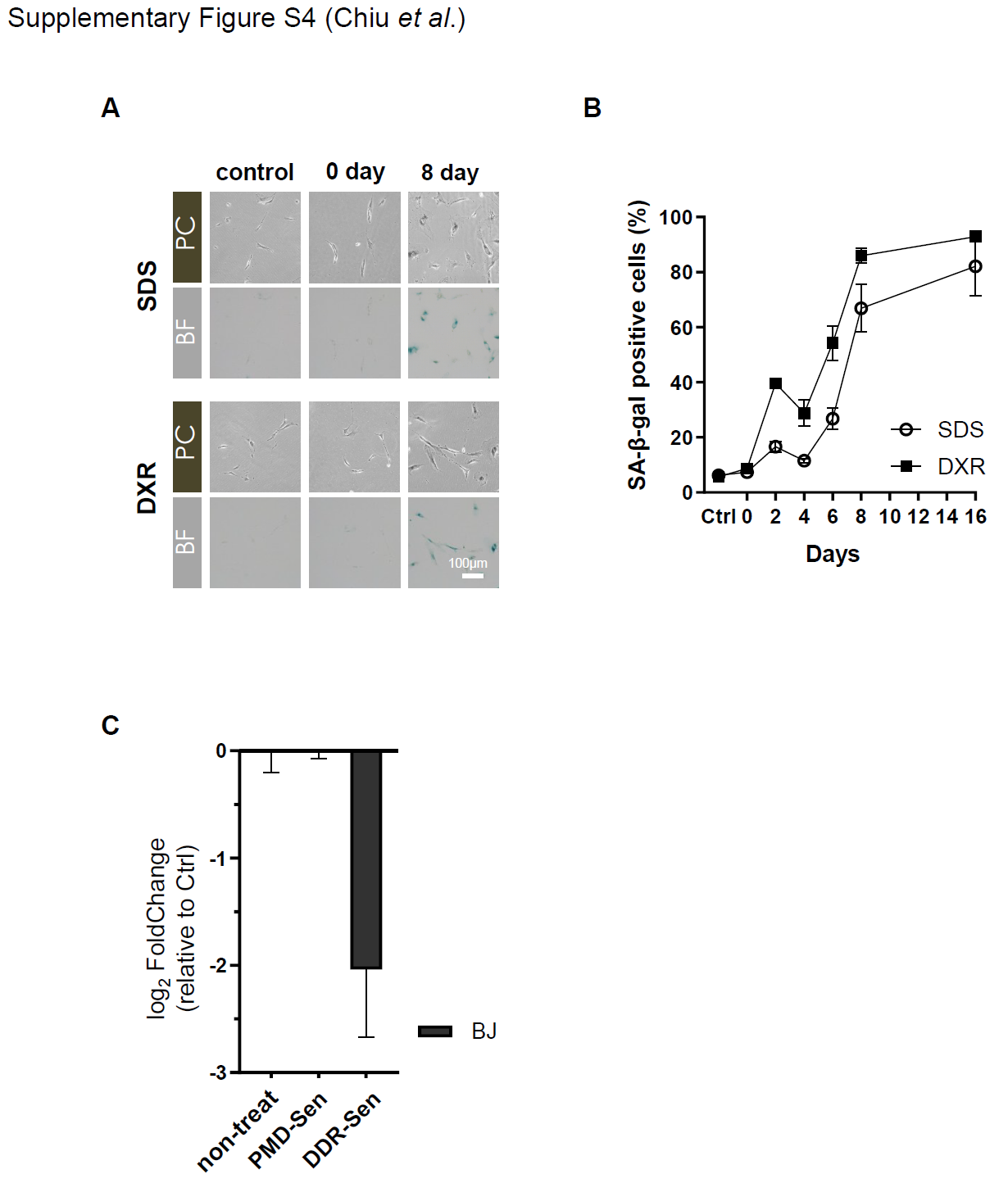

Supplement: Supplemental Material [file KRNB_A_2551299_SM1023.docx]
